# Supplementary material for: Polyurethane/Liquid Crystal Microfibers with pDNA Polyplex Loadings for the Optimal Release and Promotion of HUVEC Proliferation
Source: Pharmaceutics. 2022 Nov 17;14(11):2489. doi: 10.3390/pharmaceutics14112489 (PMC9697111; doi:10.3390/pharmaceutics14112489)
Supplement: Supplementary file 1 [file pharmaceutics-14-02489-s001.zip › pharmaceutics-1967577-supplementary.pdf]

Rabbit Model

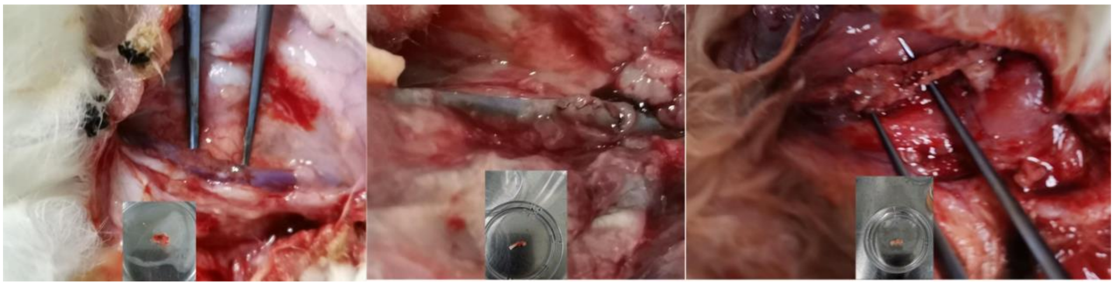

Figure S1. Photos of PU, PU/LC20%, PU/LC20%/pDNA vascular patch.

After implantation of PU, PU/LC20%, PU/LC20%/pDNA vascular patch in the carotid artery of rabbits, the wounds of the control group and the experimental group were not inflamed and healed in a short time. All of them had returned to normal activities and diet, and obvious pathological change even death did not appear in the long-term life process.

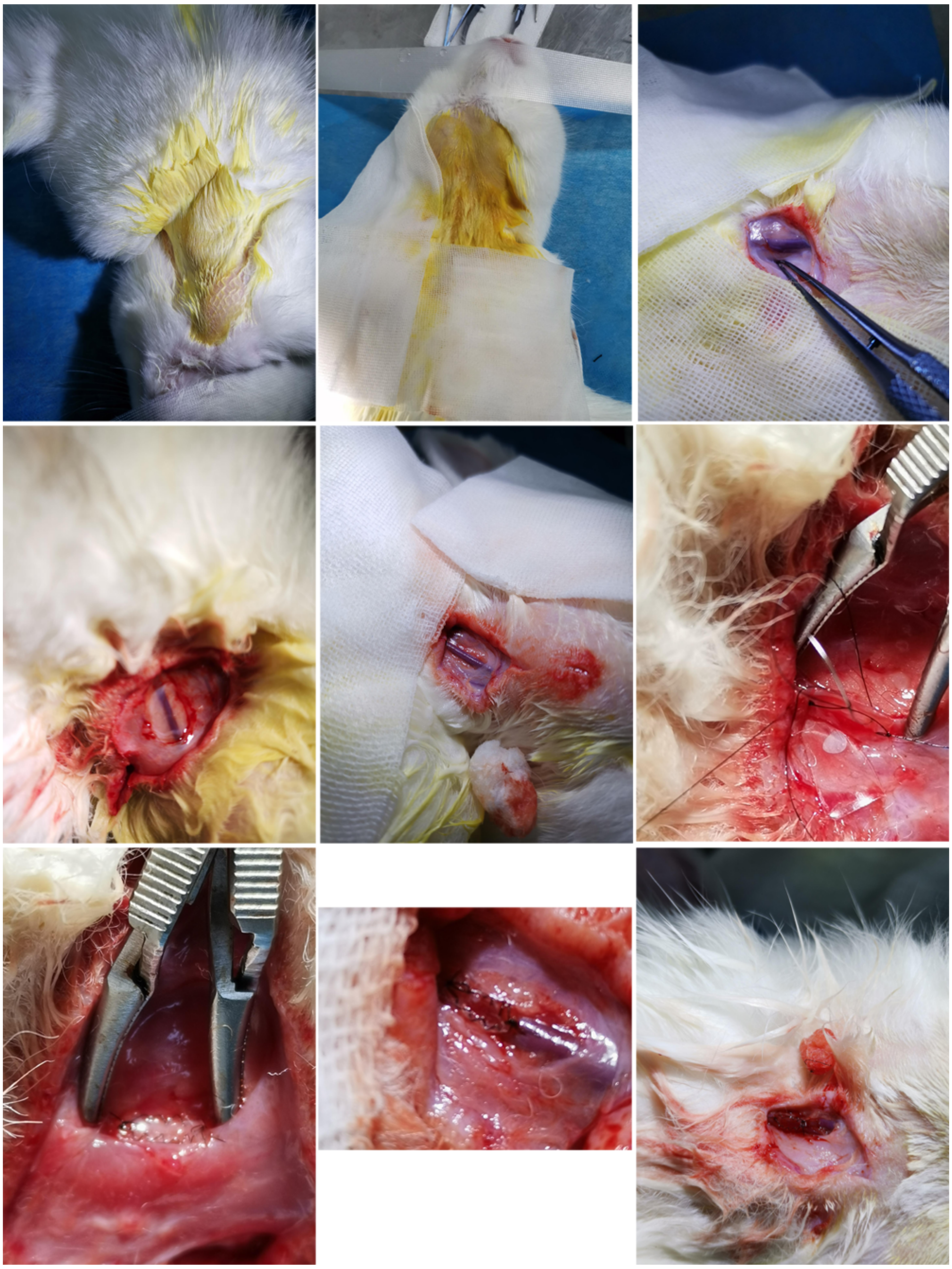

Figure S2. Photos of the surgical procedure.
